# TNFα Signals via p66^Shc^ to Induce E-Selectin, Promote Leukocyte Transmigration and Enhance Permeability in Human Endothelial Cells

**DOI:** 10.1371/journal.pone.0081930

**Published:** 2013-12-02

**Authors:** Luigi Laviola, Maura Roberta Orlando, Maria Angela Incalza, Cristina Caccioppoli, Mariangela Melchiorre, Anna Leonardini, Angelo Cignarelli, Federica Tortosa, Rossella Labarbuta, Sabina Martemucci, Consiglia Pacelli, Tiziana Cocco, Sebastio Perrini, Annalisa Natalicchio, Francesco Giorgino

**Affiliations:** 1 Department of Emergency and Organ Transplantation – Section of Internal Medicine, Endocrinology, Andrology and Metabolic Diseases, University of Bari Aldo Moro, Bari, Italy; 2 Department of Medical Biochemistry, Biology and Physics, University of Bari Aldo Moro, Bari, Italy; University of Illinois at Chicago, United States of America

## Abstract

Endothelial cells participate in inflammatory events leading to atherogenesis by regulating endothelial cell permeability via the expression of VE-Cadherin and β-catenin and leukocyte recruitment via the expression of E-Selectins and other adhesion molecules. The protein p66^Shc^ acts as a sensor/inducer of oxidative stress and may promote vascular dysfunction. The objective of this study was to investigate the role of p66^Shc^ in tumor necrosis factor TNFα-induced E-Selectin expression and function in human umbilical vein endothelial cells (HUVEC). Exposure of HUVEC to 50 ng/ml TNFα resulted in increased leukocyte transmigration through the endothelial monolayer and E-Selectin expression, in association with augmented phosphorylation of both p66^Shc^ on Ser^36^ and the stress kinase c-Jun NH_2_-terminal protein kinase (JNK)-1/2, and higher intracellular reactive oxygen species (ROS) levels. Overexpression of p66^Shc^ in HUVEC resulted in enhanced p66^Shc^ phosphorylation on Ser^36^, increased ROS and E-Selectin levels, and amplified endothelial cell permeability and leukocyte transmigration through the HUVEC monolayer. Conversely, overexpression of a phosphorylation-defective p66^Shc^ protein, in which Ser^36^ was replaced by Ala, did not augment ROS and E-Selectin levels, nor modify cell permeability or leukocyte transmigration beyond those found in wild-type cells. Moreover, siRNA-mediated silencing of p66^Shc^ resulted in marked reduction of E-Selectin expression and leukocyte transmigration. In conclusion, p66^Shc^ acts as a novel intermediate in the TNFα pathway mediating endothelial dysfunction, and its action requires JNK-dependent phosphorylation of p66^Shc^ on Ser^36^.

## Introduction

Endothelial dysfunction plays a major role in the pathogenesis of vascular damage [1]. Typically, the functional impairment of the endothelium induced by metabolic factors and/or cytokines precedes the development of morphological alterations [1,2], and is documented at a biochemical level by the abnormal expression of endothelial cell-specific genes [3]. In vitro, exposure of human endothelial cells to the pro-inflammatory cytokine TNFα results in the activation of the NF-κB [4] and MAP kinase signaling pathways [5], and expression of adhesion molecules and other mediators [6,7], which increase endothelial cell permeability [8], locally recruit circulating leukocytes and promote diapedesis through the endothelial layer, thus initiating the atherosclerotic cascade [1].

E-Selectin is a Ca^2+^-dependent cell surface glycoprotein that recruits leukocytes under proinflammatory conditions [8], and is transcriptionally induced in response to inflammatory cytokines, such as IL-1β and TNFα [9]. Elevated cellular levels of E-Selectin have been documented in various diseases associated with a pro-inflammatory condition, including diabetes, atherosclerosis, rheumatoid arthritis, and cancer [10-12]. Conversely, therapeutic strategies, which reduce vascular injury, result in significant down-regulation of E-Selectin gene expression [13-15]. 

Tight intercellular junctions connecting endothelial cells are required to prevent the vascular contents from leaking into surrounding tissue space [16]. The interaction between vascular endothelial(VE)-cadherin [17], a type-II endothelial-restricted classical cadherin, and β-catenin [18] is critical for cell-cell adhesion and cytoskeleton anchoring [19]. Disruption of the adherent junctions at the level of VE-cadherin and β-catenin is an important mechanism leading to microvascular hyperpermeability [20]. Under inflammatory conditions, the increased permeability is associated with downregulation of VE–cadherin [21]. Enhanced endothelial cell permeability associated with increased β-catenin expression and disruption of the VE-cadherin/β-catenin complexes has been observed in human endothelial cells exposed to a proinflammatory milieu [22]. Thus, defining the mechanisms regulating E-Selectin expression, endothelial cell permeability and leukocyte transmigration may increase our understanding of the development of vascular damage and may potentially identify new therapeutic targets for cardiovascular disease.

The mammalian *Shc* locus encodes for three different ShcA adaptor proteins with respective M_r_ of 46, 52, and 66 kDa. Phosphorylation of the 66-kDa isoform, p66^Shc^, on Ser^36^ has been associated with negative regulation of the p46/52^Shc^ complex, activation of oxidative stress, and increased cellular apoptosis [23-25]. Genetic deletion of p66^Shc^ in the mouse results in reduced systemic and cellular stress and increased lifespan [24]. In light of its pivotal role as a cellular stress sensor, several studies have investigated the pathophysiological contribution of p66^Shc^ to vascular damage and cardiovascular diseases. p66^Shc^ knockout mice are protected from high fat diet-induced atherosclerosis due to decreased oxidative stress and formation of foam cells [26,27], as well as from diabetes-induced endothelial dysfunction and diabetic glomerulopathy. Interestingly, p66^Shc^ protein levels appear to be increased in the aorta and renal cortex of experimental models of diabetes and in circulating leucocytes from diabetic patients [27,28]. However, the mechanisms by which p66^Shc^ may promote atherogenesis are still largely unknown and the role of p66^Shc^ in cytokine-induced endothelial dysfunction has not been addressed. 

In this study, we provide evidence that p66^Shc^, through its Ser^36^ phosphorylation, mediates TNFα-induced endothelial cell permeability by disrupting the cadherin–catenin complex and increases leukocyte transmigration through the HUVEC monolayer by increasing E-Selectin expression levels.

## Materials and Methods

### Cell cultures

HUVEC and HL-60 cells were purchased from ATCC (Manassas, VA). HUVEC were grown on six-well plates to confluence at 37°C in a humidified incubator gassed with 5% CO_2_, in F12 Kaighn’s medium (GIBCO, Palo Alto, CA), supplemented with 10% Foetal Bovine Serum (GIBCO, Palo Alto, CA), 100 IU/ml penicillin, 100 µg/ml streptomycin (LONZA, MD, Iquique, Chile), non-essential amino acids (GIBCO Invitrogen, Paisley, UK), 25 mg/ml Endothelial Cell Growth Supplement (SIGMA-ALDRICH, St Louis, MO), and 0.1 mg/ml heparin (SIGMA-ALDRICH, St Louis, MO). HUVEC were treated with the JNK inhibitors SP600125 (30 µM for 2 h) (SIGMA, St Louis MO) or JNKi peptide [29] (10 mg/ml for 2 h), the MEK inhibitor PD98059 (30 μM or 50 μM for 2 h) (Calbiochem, La Jolla, CA), the p38 MAPK inhibitor SB203580 (15 μM or 30 μM for 1 h) (Calbiochem, La Jolla, CA), the PKC-β inhibitor LY333531 (200 nM for 1 h or 24 h) (R&D Systems, Abingdon, UK) or the antioxidant agent N-acetyl-cysteine (NAC, 10 mM for 2 h) (Sigma Aldrich, St. Louis, MO) before treatment with TNFα (R&D Systems, Abingdon, UK). The NADPH oxidase (Nox)-inhibitor 3-benzyl-7-(2-benzoxazolyl)thio-1,2,3-triazolo[4,5-d]pyrimidine (VAS2810, 5 µM), and the complex I (NADH dehydrogenase) inhibitors rotenone (10 µM) and thenoyltrifluoroacetone (TTFA, 10 µM) (purchased from SIGMA-ALDRICH, St Louis, MO) were coincubated with TNFα for 0.5 h. The JNKi peptide sequence, *5*(*6*)*-FAM-*GRKKRRQRRRPPRPKRPTTLNLFPQVPRSQDT-*COOH*, was synthetized by Primm (Milan, Italy) and linked to a FITC fluorochrome to visualize its cell entry and accumulation. The promyelocytic cell line HL-60 was cultured in Iscove’s Modified Dulbecco’s Medium (ATCC, Manassas, VA) with 100 IU/ml penicillin, 100 μg/ml streptomycin (LONZA, MD, Iquique, Chile), and 10% of FBS (GIBCO, Palo Alto, CA). Cell viability was assessed by trypan blue dye (SIGMA-ALDRICH, St Louis, MO) exclusion. Cell number was calculated using Scepter^TM^ Handheld Automated Cell Counter (Millipore, Bedford, MA).

### Adenoviral transfection studies

HUVEC expressing increased levels of the p66^Shc^ protein were obtained as described previously [30]. Briefly, the gene of interest was cloned into the shuttle vector pAdTrack-CMV, then co-transformed into *E. coli* BJ5183 cells with the adenoviral backbone plasmid pAdEasy-1, and transfected into the adenovirus packaging cell line QBI293A. Scalar doses of adenovirus-containing culture medium were used to biologically define the optimal HUVEC infection dose (>90% of infected cells). To obtain HUVEC overexpressing a dominant-negative p66^Shc^ mutant, cells were transfected with an adenoviral construct carrying a Ser^36^ to Ala^36^ mutation. An empty adenovirus was used as control for the infection (mock).

### siRNA transfection studies

Two independent siRNAs were used to obtain a selective reduction of p66^Shc^ mRNA and protein expression: siRNA#1 (5’-AUGAGUCUCUGUCAUCGCUTT-3’; 5’-AGCGAUGACAGAGACUCAUTC-3’) [31], and siRNA#2 (5’-UGAGUCUCUGUCAUCGCUGTT-3’; 5’-CAGCGAUGACAGAGACUCATT-3’. Both siRNA were designed using the *siRNA Target Finder* software and synthesized by Qiagen (Hilden, Germany). Two independent siRNAs were used to obtain a selective reduction of E-Selectin expression: siRNA#1 [32] was synthesized by Qiagen and siRNA#2 [33] was purchased by Santa Cruz Biotechnology Inc. (sc-29296, Santa Cruz, CA). Control, non-silencing fluorescently labeled siRNA was obtained from Qiagen (AllStars Negative Control siRNA, Hilden, Germany). Cell transfection was achieved using Lipofectamine™ (Invitrogen, Carlsbad, CA), according to the manufacturer’s instructions. Selective reduction of p66^Shc^ gene and protein expression was verified by real-time RT-PCR (qPCR) two and three days following transfection, respectively.

### Immunoblotting

Cells were lysed in 50 mM HEPES, pH 7.5, containing 150 mM NaCl, 1 mM MgCl_2_, 1 mM CaCl_2_, 4 mM EDTA, 1% Triton X-100, 10% glycerol, 10 mM NaF, and 10 mM NaPP, supplemented with 4% protease inhibitor cocktail (Roche Diagnostics, Indianapolis, IN), and cleared by centrifugation. Protein concentration was determined by the Bradford assay (Bio-Rad, Hercules, CA). Equal protein samples (30-80 µg) were separated on SDS-PAGE gels and electro-transferred onto Hybond-P polyvinylidene difluoride filters (Amersham Life Science, Arlington Heights, IL). The filters were then probed with the specific primary antibodies, and the immune-reactive bands visualized with horseradish peroxidase (HRP)-conjugated goat anti-mouse IgG, goat anti-rabbit IgG, or donkey anti-sheep IgG (H+L) (Bio-Rad, Hercules, CA), as appropriate, using an ECL Plus Western Blotting Detection System (Amersham Life Science, Arlington Heights, IL), and quantified by densitometric analysis using the Versadoc imaging system (Bio-Rad, Hercules, CA).

### Antibodies

Polyclonal antibody against Shc was purchased from Transduction Laboratories (Lexington, KY); antibody against p66^Shc^-phospho-Ser^36^ was from Calbiochem (La Jolla, CA); antibodies against MAP kinases (ERK-1/2) were from Zymed Laboratories (San Francisco, CA), polyclonal antibodies against phospho-p42/p44 MAP kinases (Thr^202^/Tyr^204^), phospho-SAPK/JNK kinases (Thr^183^/Tyr^185^), SAPK/JNK kinases, c-Jun-phospho-Ser^63^, c-Jun and phospho-p38 kinases (Thr^180^/Tyr^182^) were purchased from Cell Signaling Technology (Beverly, MA); polyclonal antibody against phospho-IκBα (Ser^32^), GAPDH and β-actin were from Santa Cruz Biotechnology Inc. (Santa Cruz, CA); polyclonal antibody against E-Selectin was from R&D Systems (Abingdon, United Kingdom), polyclonal antibody against β-catenin was from Abcam (Cambridge, United Kingdom), and polyclonal antibody against VE-cadherin was from Millipore (Bedford, MA).

### qRT-PCR

Total RNA was isolated from HUVEC using the RNeasy Mini Kit (Qiagen, Hilden, Germany). Genomic DNA contamination was eliminated by DNase digestion (Qiagen, Hilden, Germany), and cDNA was obtained using the High Capacity cDNA Reverse Transcription Kit (Applied Biosystems, Weiterstadt, Germany). Primers for E-Selectin, p66^Shc^, and β-actin were designed using Primer Express 3.0 (Applied Biosystems, Weiterstadt, Germany). Primer sequences were designed as follows: E-Selectin_For: 5’-GAAGGATGGACGCTCAATGG-3’ and E-Selectin_Rev: 5’-TGGACTCAGTGGGAGCTTCAC-3’; p66^Shc^_For: 5’-CCCCCAAGCCCAAGTACAA-3’; p66^Shc^_Rev: 5’-GACCCAGAAGCCCCTTCCT-3’; β-actin_For: 5’-TGGATCAGCAAGCAGGAGTATG-3’ and β-actin_Rev: 5’-GCATTTGCGGTGGACGAT-3’. The PCR reactions were carried out in an ABI PRISM 7500 System (Applied Biosystems, Weiterstadt, Germany). The PCR reactions were carried out under the following conditions: 50°C for 2 min, 95°C for 10 min, 40 cycles at 95°C for 15 sec, and 60°C for 1 min. Relative gene expression levels were determined by analyzing the changes in SYBR green fluorescence during qRT-PCR using the ΔΔCt method. To confirm amplification of specific transcripts, melting curve profiles were produced at the end of each reaction. The mRNA level of each gene was normalized using β-actin as internal control.

### Immunofluorescence analyses

Wild-type HUVEC and HUVEC infected with the various adenoviral constructs were grown on coverslips in complete medium under basal conditions or after stimulation with TNFα, and then treated for confocal analysis, as follows. HUVEC were fixed with 3.7% formaldehyde, permeabilized, and incubated with the NF-κB (1:50, Santa Cruz Biotechnology Inc, CA), β-catenin (1:2000, Abcam, Cambridge, United Kingdom), or VE-cadherin antibody (1:57, Millipore, Bedford, MA) for 90 min at room temperature followed by the secondary Alexa^546^ Fluor anti-mouse or anti-rabbit antibody for 1 h at room temperature (1:300, Molecular Probes, Eugene, OR) in PBS containing 3% BSA, respectively. Coverslips were mounted on glass slides with Vectashield (VECTOR laboratories, Burlingame, CA). Images were acquired on a Leica DM IRE2 & DM IRB confocal microscope (Leica Microsystems, Heerbrugg, Switzerland). Data were quantified using the Cell Profiler Software (www.cellprofiler.org).

### ROS analyses

ROS production was detected through the evaluation of dihydroethidium (DHE) oxidation using a Jasco FP6200 spectrofluorimeter (Jasco, Easton, MD). Cells were incubated with 20 mM DHE for 0.5 h at 37°C in a serum-free medium in the dark, then washed with PBS, collected by trypsinization and resuspended in assay buffer (100 mM potassium phosphate, pH 7.4, 2 mM MgCl_2_), using an aliquot for protein determination. The fluorescence increase (480 nm excitation and 567 nm emission wavelength) caused by the ROS-dependent oxidation of DHE was expressed as arbitrary units normalized by cell protein content.

### Leukocyte transmigration test

Colorimetric QCM^TM^ Leukocyte Transendothelial Cell Migration Test (Millipore, Bedford, MA) was used to investigate the migration of HL60 through a HUVEC monolayer. HUVEC (10^5^ cells) were seeded onto fibronectin pre-coated cell culture inserts, incubated for 48 h with E-Selectin siRNA#1 (50 nM), E-Selectin siRNA#2 (100 nM), p66^Shc^ siRNA#1 (100 nM), p66^Shc^ siRNA#2 (50 nM), Ad/p66^Shc^, Ad/p66^Shc^ *Ala* ^*36*^, or Ad/ mock, and grown to confluency for 72 h. HL-60 (2x10^5^ cells) were added on the endothelial cell layer and left to migrate for 18 h at 37°C. Studies were carried out in the absence or presence of 50 ng/ml TNFα as positive control. Migrated cells were stained and measured by fluorimetry at OD 450 nm after 4 h of incubation at 37°C. The measurements were repeated three times every 15 min.

### FITC-dextran permeability assay

HUVEC were grown on transwell permeable supports (pore size, 1 µm; Millipore, Bedford, MA). Control cells and adenovirus-infected cells, overexpressing wild-type or mutant p66^Shc^, were incubated with 50 ng/ml TNFα for 4 h or left untreated. FITC-dextran was added to the upper chamber for 20 min. The amount of FITC-dextran diffused into the bottom chamber was determined by measuring the fluorescence at 485 nm and 535 nm of excitation and emission, respectively, and expressed as arbitrary units.

### Statistical analysis

Data are presented as mean ±SE of at least three independent experiments, unless differently specified in the Figure legend, and are expressed as percentage of control values, as appropriate. Normal distribution of data was assessed by the Kolmogorov–Smirnov test (*P*>0.05). Statistical analysis was performed by Student’s t test or one-way ANOVA with Tukey’s multiple comparison test, as appropriate, using Minitab® 15.1. Significance was assumed at a P value of less than 0.05.

## Results

### TNFα action and signaling in HUVEC

**Figure 1:**
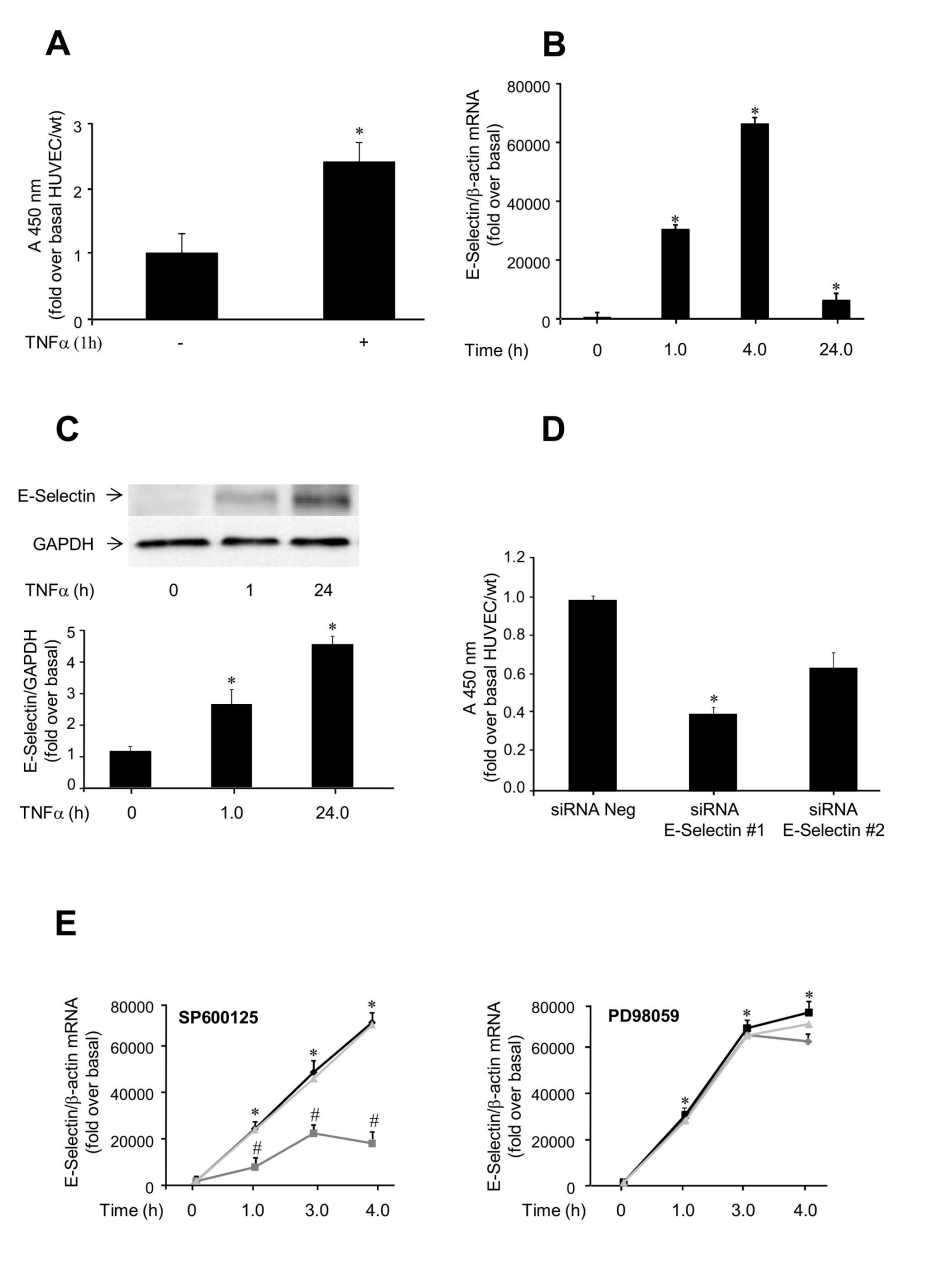
Effects of TNFα on endothelial activation in HUVEC. Cells were incubated with 50 ng/ml TNFα for the indicated times or left untreated. *A*. Leukocyte transmigration test. HL-60 cells were added to HUVEC/wt, stimulated with 50 ng/ml TNFα for 4 h, and left to migrate for 18 h at 37°C. Migrated cells were stained and measured by OD at 450 nm. Data represents the mean of triplicates from two independent experiments, and are normalized to control. *P<0.05 vs. no TNFα. *B*. TNFα regulation of E-Selectin mRNA levels. E-Selectin mRNA levels were evaluated by qRT-PCR, and normalized using β-actin as internal control. *C*. TNFα regulation of E-Selectin protein levels. E-Selectin protein levels were evaluated by immunoblotting, using GAPDH as internal control. *D*. Leukocyte transmigration test. HL-60 cells were added to control HUVEC (treated with negative siRNA) and HUVEC treated with E-Selectin siRNA#1 (50 nM) or siRNA#2 (100 nM) for 48 h, and left to migrate for 18 h at 37°C. Studies were carried out under basal conditions. Migrated cells were stained and measured by OD at 450 nm. Data represents the mean of triplicates of one experiment and are normalized to control. *P<0.05 vs. negative siRNA. *E*. Effects of SP600125 (left) and PD98059 (right) on TNFα-induced E-Selectin mRNA expression. Cells were pre-treated with 30 mM JNK or MEK inhibitor, respectively, for 2 h and then exposed 50 ng/ml TNFα for the indicated times (untreated cells, black; inhibitor-treated cells, grey; DMSO-treated cells, light grey). E-Selectin mRNA levels were evaluated by qRT-PCR, using β-actin as internal control. *P<0.05 vs. basal; ^#^ *P*<0.05 vs. TNFα-stimulated cells.

Leukocyte transmigration through the endothelial monolayer, which represents a marker of endothelial activation, was significantly increased after exposure of HUVEC to TNFα for 1 h (*P*<0.05 vs. basal; Figure 1A). E-Selectin mRNA levels, measured by qRT-PCR, were also significantly increased by TNFα, reaching a peak at 4 h (*P*<0.05 vs. basal; Figure 1B). In addition, E-Selectin protein levels were time-dependently increased by TNFα, and persisted elevated up to 24 h (*P*<0.05 vs. basal; Figure 1C). Selective inhibition of E-Selectin expression following transfection of a specific siRNA (Figure S1A) resulted in significantly decreased leukocyte transmigration (*P*<0.05 vs. control; Figure 1D), indicating a major role of this adhesion molecule in endothelial activation.

Exposure to TNFα induced the phosphorylation of the stress kinases JNK-1 and JNK-2 in HUVEC, with a significant activation peak at 0.5 h (*P*<0.05 vs. untreated cells; Figure S1B). Similarly, ERK-1/2 was also rapidly activated upon exposure to TNFα. ERK-1/2 phosphorylation increased approximately 2-fold both 0.5 h and 1 h following stimulation with TNFα (*P*<0.05 vs. basal), and then gradually declined (Figure S1C). To investigate the role of JNK in TNFα-induced E-Selectin expression, HUVEC were pretreated with SP600125, prior to challenge with TNFα. Under these conditions, the TNFα-induced increase in E-Selectin mRNA levels was reduced by 85% (*P*<0.05 vs. TNFα-treated cells; Figure 1E). By contrast, preincubation with the MEK inhibitor PD98059 (30 µM) did not alter the effect of TNFα on E-Selectin mRNA (Figure 1E). Similar results were obtained also using higher concentrations of PD98059 (50 µM, data not shown). The TNFα-induced increase in E-Selectin protein levels was also inhibited by the JNK inhibitors SP600125 and the JNKi peptide and not by the MEK inhibitor, respectively (Figure S2, *A-D*). Altogether, these findings indicate that TNFα stimulates the increase of E-Selectin gene expression via JNK but not ERK signaling in HUVEC.

### Effects of TNFα on p66^Shc^ activation in HUVEC

**Figure 2:**
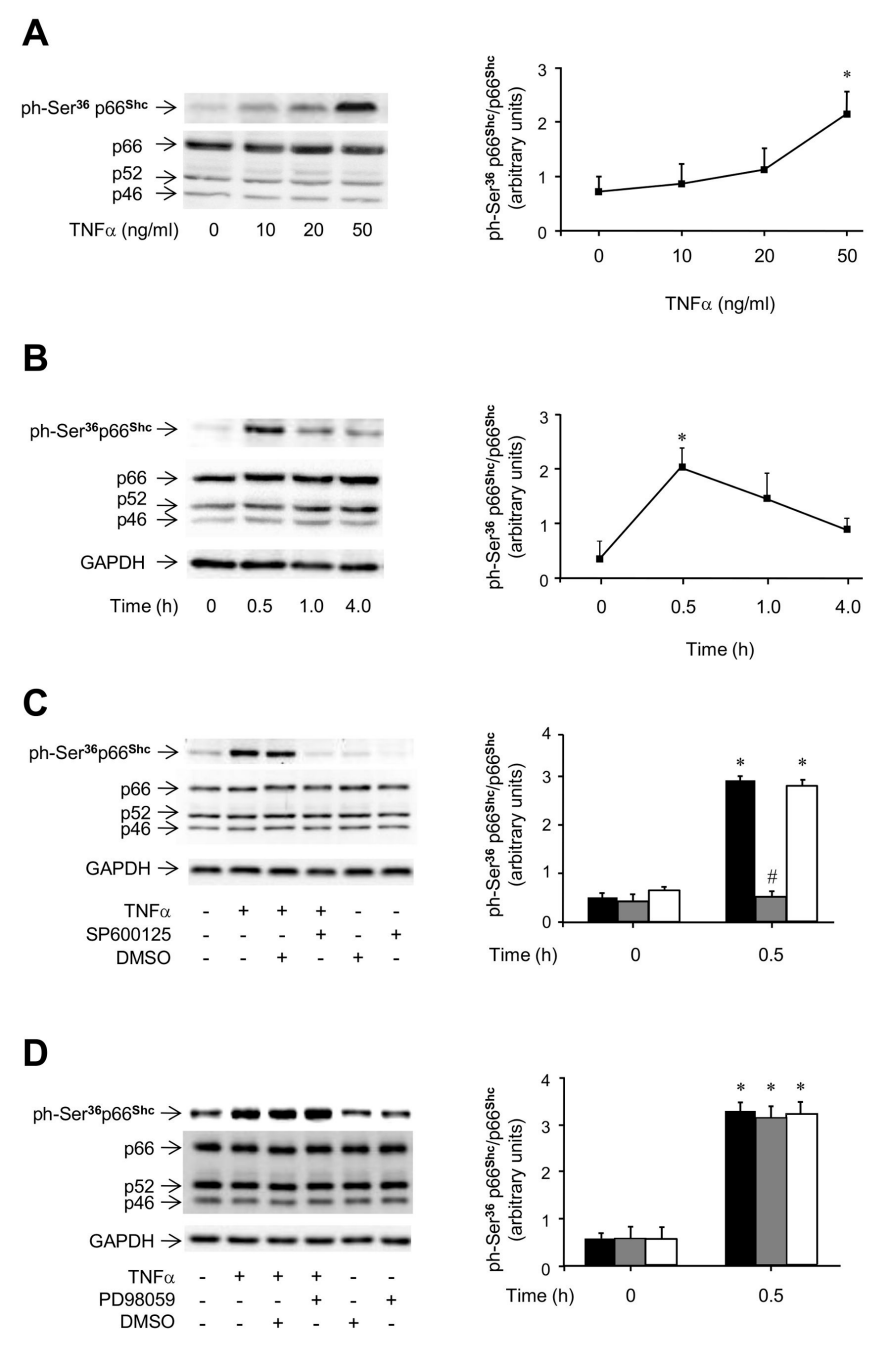
TNFα-induced phosphorylation of p66^Shc^ on Ser^36^ in HUVEC. *A*. Dose-response studies. Cells were incubated with TNFα for 0.5 h at the indicated doses or left untreated. Representative immunoblots of p66^Shc^ phosphorylation on Ser^36^ (top left) and Shc protein content (bottom left), and ratio of phosphorylated to total p66^Shc^ protein (right). Cell lysates were analyzed by immunoblotting with specific antibodies. *B*. Time-course studies. Cells were incubated with 50 ng/ml TNFα for the indicated times or left untreated. Representative immunoblots of p66^Shc^ phosphorylation on Ser^36^ (top left) and Shc protein content (bottom left), and ratio of phosphorylated to total p66^Shc^ protein (right). *C*. Effects of the JNK inhibitor SP600125 on TNFα-induced phosphorylation of p66^Shc^ on Ser^36^. Cells were pre-treated with 30 mM SP600125 for 2 h and then exposed to 50 ng/ml TNFα for 0.5 h. Representative immunoblots of p66^Shc^ phosphorylation on Ser^36^ (top left) and Shc protein content (middle left), and ratio of phosphorylated to total p66^Shc^ protein (*right*; untreated cells, black bars; inhibitor-treated cells, grey bars; DMSO-treated cells, white bars). *D*. Effects of the MEK inhibitor PD98059 on TNFα-induced phosphorylation of p66^Shc^ on Ser^36^. Cells were pre-treated with 30 mM PD98059 for 2 h and then exposed to 50 ng/ml TNFα for 0.5 h. Representative immunoblots of p66^Shc^ phosphorylation on Ser^36^ (top left) and Shc protein content (middle left), and ratio of phosphorylated to total p66^Shc^ protein (*right*; untreated cells, black bars; inhibitor-treated cells, grey bars; DMSO-treated cells, white bars). GAPDH protein content was used as loading control. *P<0.05 vs. basal; ^#^ *P*<0.05 vs. controls.

To assess the potential involvement of p66^Shc^ in TNFα signaling, HUVEC were exposed to increasing doses of TNFα (0-50 ng/ml), and p66^Shc^ phosphorylation on Ser^36^ was evaluated. Exposure to TNFα induced a dose-dependent increase in p66^Shc^ Ser-phosphorylation, which was augmented 2-fold with 50 ng/ml of TNFα (*P*<0.05 vs. untreated cells; Figure 2A). Time-course analysis of p66^Shc^ phosphorylation in response to TNFα showed a 3-fold significant increase that occurred after 0.5 h (*P*<0.05 vs. basal; Figure 2B). The contribution of JNK and ERK activation to the TNFα-induced Ser-phosphorylation of p66^Shc^ was then studied using the SP600125 and PD98059 inhibitors, respectively. When cells were pretreated with SP600125, the TNFα effect on p66^Shc^ Ser-phosphorylation was greatly reduced (*P*<0.05 vs. TNFα-treated cells; Figure 2C). By contrast, treatment with PD98059 did not affect the ability of TNFα to induce p66^Shc^ phosphorylation on Ser^36^ (Figure 2D). When cells were pretreated with the p38 MAPK inhibitor SB203580 or the PKC-β inhibitor LY333531, respectively, the TNFα effect on p66^Shc^ Ser-phosphorylation was partially impaired (P<0.05 vs. controls; Figure S3A and B). Thus, these results suggests that JNK, and partially p38 and PKC-β, but not ERK, are involved in the ability of TNFα to rapidly stimulate p66^Shc^ phosphorylation on Ser^36^ in HUVEC.

### Effects of p66^Shc^ overexpression on TNFα action in HUVEC

**Figure 3:**
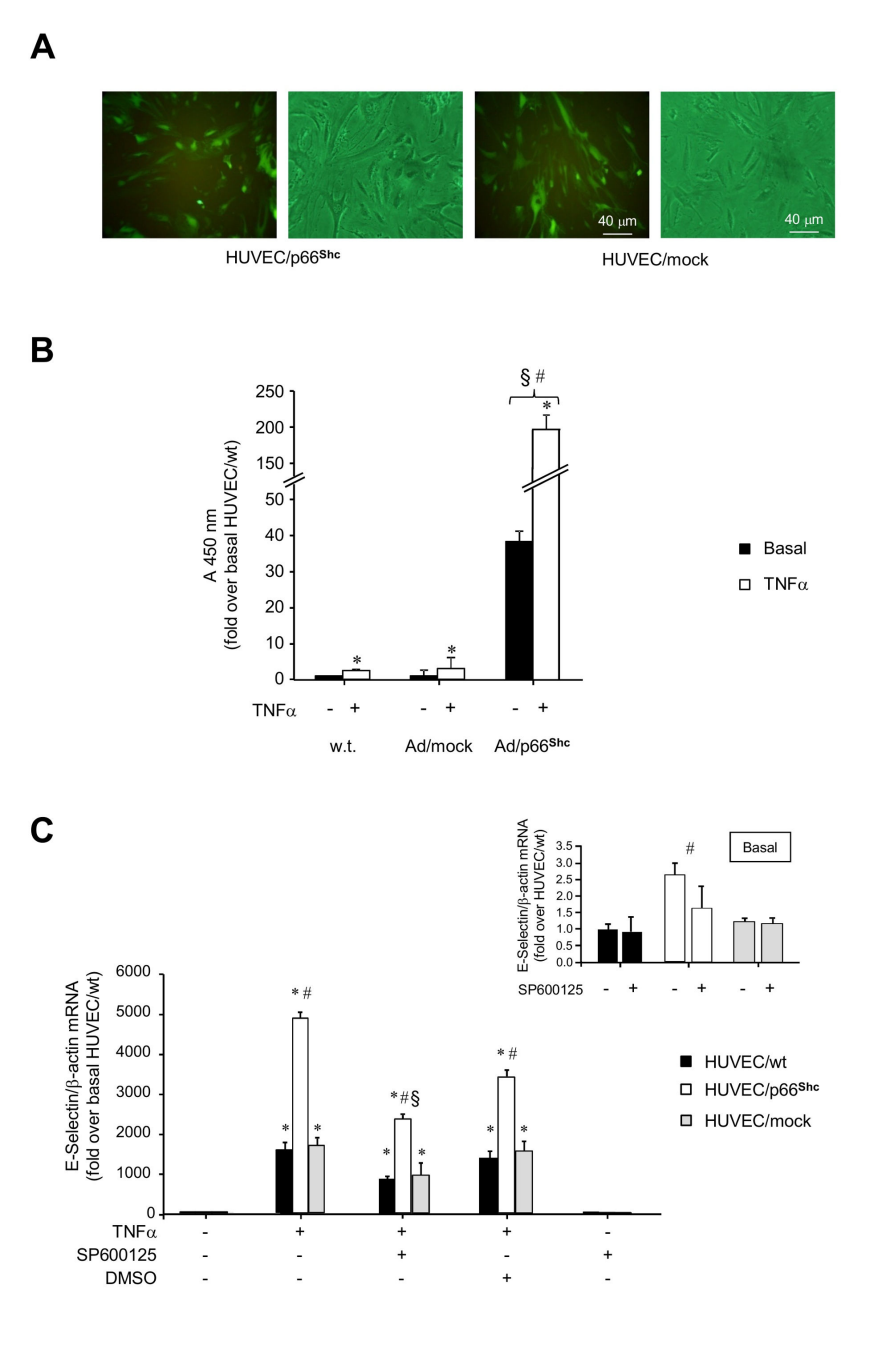
Effects of p66^Shc^ overexpression on endothelial activation in HUVEC. A. Representative images of HUVEC/p66^Shc^ and HUVEC/mock. Cells were infected with the adenoviral constructs and analyzed by fluorescent microscopy. The green staining identifies cells expressing the green fluorescent protein (GFP) encoded by the recombinant adenovirus. Cellular morphology was evaluated by optical microscopy. B. Leukocyte transmigration test. HL-60 cells were added to HUVEC/wt and HUVEC transduced with Ad/p66^Shc^ or Ad/mock for 48 h, and left to migrate for 18 h at 37°C. Studies were carried out under basal conditions or following stimulation with 50 ng/ml TNFα for 4 h. Migrated cells were stained and measured by OD at 450 nm. Data represents the mean of triplicates from two independent experiments, and are normalized to basal HUVEC/wt. *P<0.05 vs. no TNFα; ^§^P<0.05 vs. controls. C. Effects of p66^Shc^ overexpression on E-Selectin mRNA levels in HUVEC. HUVEC/wt (filled bars), HUVEC/p66^Shc^ (open bars), and HUVEC/mock (grey bars) were treated with 50 ng/ml TNFα for 1 h or left untreated. When indicated, cells were pre-treated with 30 mM SP600125 or DMSO for 2 h. E-Selectin gene expression was evaluated using qRT-PCR. The inset (right) shows E-Selectin mRNA levels under basal conditions. *P<0.05, TNFα-stimulated cells vs. unstimulated cells; ^#^P<0.05, HUVEC/p66^Shc^ vs. controls; ^§^P<0.05, SP600125-treated cells vs. controls.

To investigate the role of p66^Shc^ in TNFα signaling and action, HUVEC with adenovirus-mediated overexpression of p66^Shc^ (HUVEC/p66^Shc^) were obtained. HUVEC/p66^Shc^ showed normal cellular morphology, similarly to control cells (Figure 3A). Moreover, in the context of augmented p66^Shc^ expression and phosphorylation (Figure S4, *A-C*), the pattern of JNK and ERK activation was similar in HUVEC/p66^Shc^ and control cells, since JNK-1/2 phosphorylation peaked at 0.5 h (*P*<0.05 vs. untreated cells; Figure S5A), and ERK-1/2 phosphorylation was increased at 0.5 h and 1 h following exposure to TNFα (*P*<0.05 vs. untreated cells; Figure S5B). Increased p66^Shc^ expression and phosphorylation also did not apparently affect other signaling pathways, such as that involving NF-κB. In both HUVEC/wt and HUVEC/mock, NF-κB was found to be localized in the cytoplasm in the basal state and was translocated to the nucleus following TNFα stimulation (Figure S6, *A* and B), and similar results were obtained in HUVEC/p66^Shc^ (Figure S6D). In parallel, Ser^32^-IκBα phosphorylation was induced by TNFα, but not affected by p66^Shc^ overexpression (Figure S6E). Therefore, increased p66^Shc^ expression and phosphorylation was apparently not linked to or did not interfere with the ERK or NF-κB signaling pathways, and appeared to be downstream of JNK activation.

**Figure 4:**
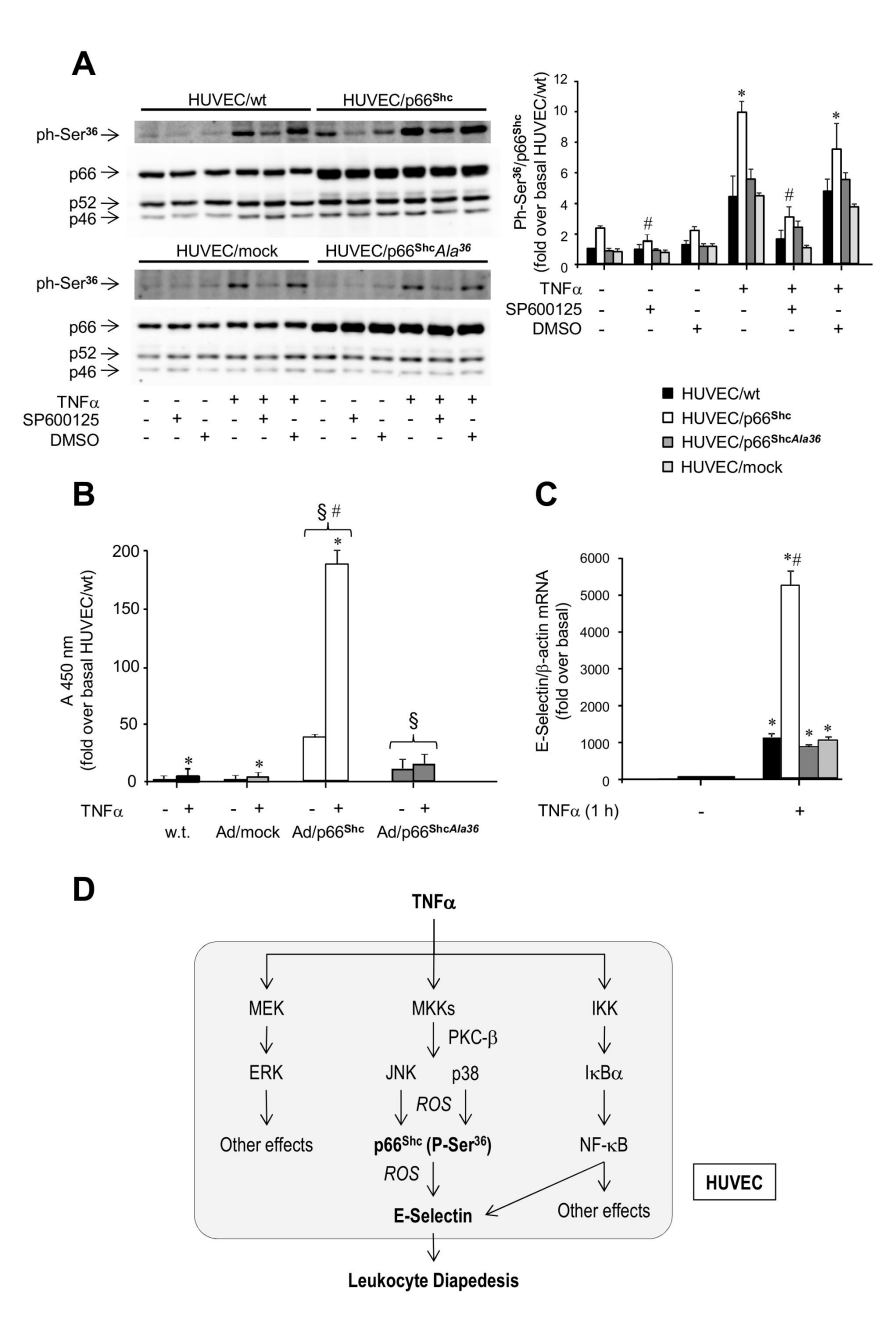
Role of Ser^36^ phosphorylation of p66^Shc^ in endothelial activation. *A*. Basal and TNFα-stimulated phosphorylation of p66^Shc^ on Ser^36^ in HUVEC overexpressing the p66^Shc^ Ala^36^ mutant (HUVEC/p66^Shc^Ala^36^). Cells were stimulated with 50 ng/ml TNFα for 0.5 h or left untreated. When indicated, cells were pre-incubated with 30 mM SP600125 or DMSO for 2 h prior to TNFα stimulation. Each pair of representative immunoblots shows p66^Shc^ phosphorylation on Ser^36^ (top left) and Shc protein content (bottom left) in HUVEC/wt, HUVEC/p66^Shc^, HUVEC/p66^Shc^Ala^36^, and HUVEC/mock, respectively. The ratio of phosphorylated to total p66^Shc^ protein in the four cell lines is also shown (*right*; HUVEC/wt, filled bars; HUVEC/p66^Shc^, open bars; HUVEC/p66^Shc^Ala^36^, grey bars; HUVEC/mock, light grey bars). *P<0.05, TNFα-stimulated HUVEC/p66^Shc^ vs. HUVEC/p66^Shc^Ala^36^; ^#^ *P*<0.05, SP600125-treated HUVEC/p66^Shc^ vs. untreated HUVEC/p66^Shc^. *B*. Leukocyte transmigration test. HL-60 cells were added to HUVEC/w.t. and HUVEC treated with Ad/p66^Shc^, Ad/p66^Shc^Ala^36^, or Ad/mock for 48 h, and left to migrate for 18 h at 37°C. Studies were carried out under basal conditions or following stimulation with 50 ng/ml TNFα for 4 h. Migrated cells were stained and measured by OD at 450 nm. Data represents the mean of triplicates from two independent experiments, and are normalized to basal HUVEC/wt. *P<0.05 vs. no TNFα; ^§^P<0.05 vs. HUVEC/wt; ^#^ *P*<0.05 vs. HUVEC/p66^Shc^Ala^36^. *C*. Effects of Ser/Ala^36^ mutation on E-Selectin mRNA levels in HUVEC. HUVEC/wt (filled bars), HUVEC/p66^Shc^ (open bars), HUVEC/*Ala* ^36^ (grey bars), and HUVEC/mock (light grey bars) were treated with 50 ng/ml TNFα for 1 h or left untreated. E-Selectin gene expression was evaluated using qRT-PCR. *P<0.05, TNFα-stimulated cells vs. unstimulated cells; ^#^ *P*<0.05, HUVEC/p66^Shc^ vs. controls. *D*. Cartoon illustrating the signaling pathway mediating the stimulatory effect of TNFα on E-Selectin gene expression in HUVEC. MKKs, MKK-7 and MKK-4; IKK, IκBα kinase; ROS, reactive oxygen species.

Importantly, though, p66^Shc^ overexpression potentiated the ability of TNFα to increase leukocyte transmigration and E-Selectin expression. Under basal conditions, leukocyte transmigration was significantly augmented (*P*<0.05 vs. controls; Figure 3B) and E-Selectin mRNA levels were moderately higher than in control cells (*P*<0.05 vs. HUVEC/wt and HUVEC/mock; Figure 3C, *inset*). However, after treatment with 50 ng/ml TNFα for 1 h, both leukocyte transmigration and E-Selectin mRNA were induced several-fold (*P*<0.01 vs. untreated HUVEC/p66^Shc^; Figure 3, B and C), and markedly higher in HUVEC/p66^Shc^ compared with control cells (*P*<0.05 vs. HUVEC/wt and HUVEC/mock; Figure 3, B and C). E-Selectin protein was also found to be increased in HUVEC/p66^Shc^ compared to control cells after exposure to TNFα (*P*<0.05 vs. HUVEC/mock after TNFα for 1 h; Figure S5C). Treatment of HUVEC/p66^Shc^ with SP600125 tended to reduce basal E-Selectin gene expression (*P*=0.081 vs. untreated HUVEC/p66^Shc^; Figure 3C, *inset*), and significantly reduced TNFα-stimulated E-Selectin mRNA (*P*<0.05 vs. HUVEC/p66^Shc^ cells treated with TNFα alone; Figure 3C), and this was paralleled by decreased p66^Shc^ Ser^36^ phosphorylation levels both under basal conditions and following TNFα stimulation (*P*<0.05 vs. controls, Figure 4A). In contrast, preincubation of HUVEC/p66^Shc^ with PD98059 had no effect on p66^Shc^ phosphorylation and did not modify basal or TNFα-induced E-Selectin mRNA levels (data not shown). 

**Figure 5:**
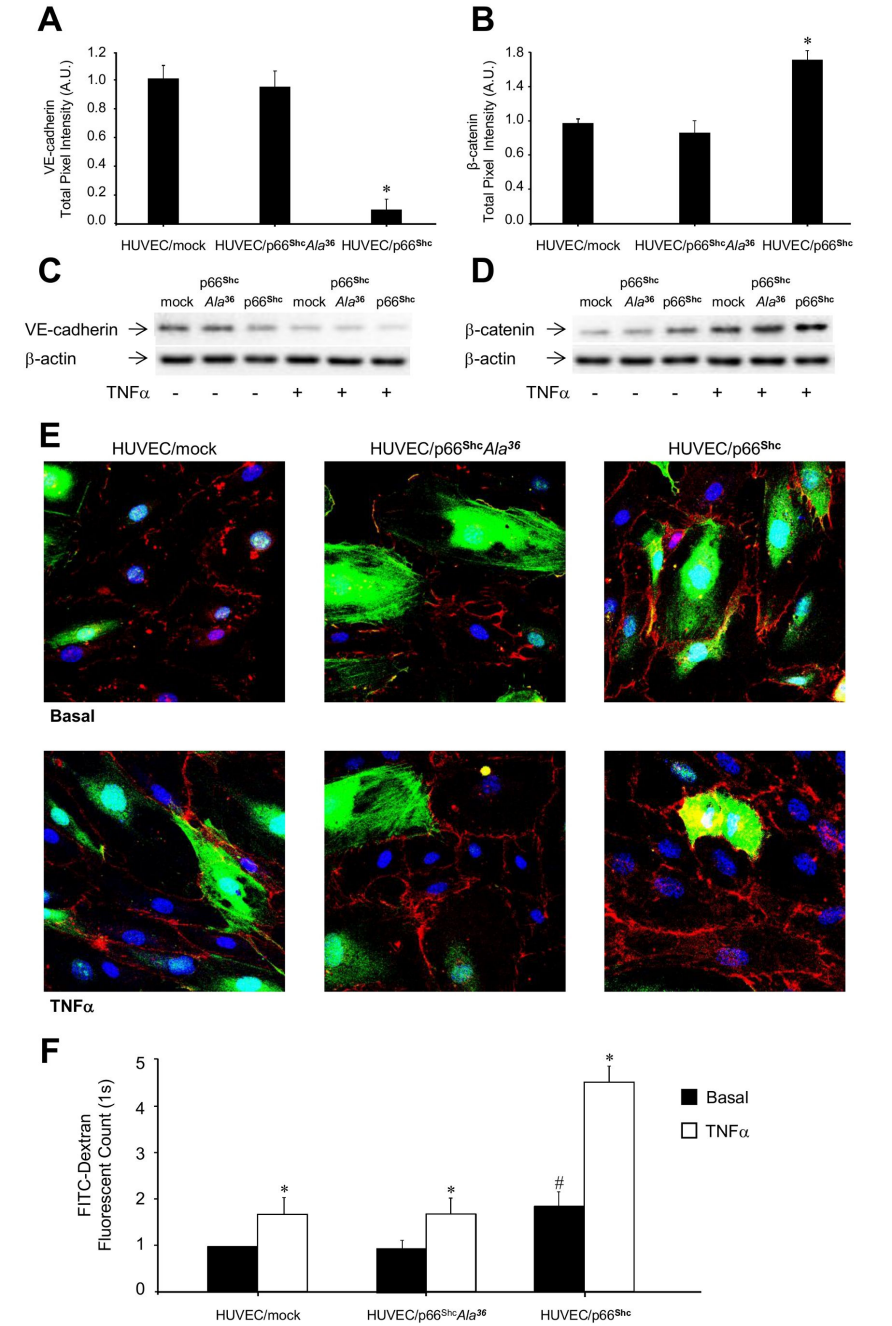
Role of p66^Shc^ in HUVEC permeability. Protein levels of VE-cadherin and β-catenin in HUVEC/mock, HUVEC/p66^Shc^, and HUVEC/p66^Shc^Ala^36^ by immunofluorescence (*A* and *B*) and immunoblotting (*C* and *D*) analyses, respectively. *E*. Representative immunofluorescence images of β-catenin expression in HUVEC/mock, HUVEC/p66^Shc^, and HUVEC/p66^Shc^Ala^36^, respectively, under basal conditions and following treatment with 50 ng/ml TNFα for 0.5 h. Adenovirus-infected cells are shown in green, β-catenin antibody staining is in red, and TOPRO-stained nuclei are in blue. *F*. Vascular permeability assay. FITC-dextran was added to the experimental cells, under basal conditions or following stimulation with 50 ng/ml TNFα for 4 h. The amount of FITC-dextran diffused into the bottom chamber was evaluated by measuring the fluorescence at 485 nm and 535 nm of excitation and emission, respectively. *P<0.05 vs. no TNFα; ^#^ *P*<0.05 vs. controls.

The effects of p66^Shc^ overexpression on Vascular Endothelial (VE)-cadherin and β-catenin expression were investigated next, to explore the potential involvement of p66^Shc^ in regulating vascular permeability. Cells with increased p66^Shc^ expression showed downregulation of VE-cadherin (Figure 5, *A* and *C*) and increased expression of β-catenin (Figure 5, *B, D* and *E*) compared to controls (HUVEC/mock). Since the VE-cadherin/β-catenin complex modulates endothelial permeability, a FITC-dextran-based assay was used to evaluate the permeability of HUVEC monolayers. Leakage of the FITC-dextran marker was found to be increased in HUVEC/p66^Shc^ compared to control HUVEC (*P*<0.05; Figure 5F). Exposure to 50 ng/ml TNFα for 4 h resulted in reduced VE-cadherin and increased β-catenin expression (Figure 5, *C-E*), and greater FITC-dextran leakage in control HUVEC and even more so in HUVEC/p66^Shc^ (*P*<0.05 vs. control HUVEC; Figure 5F). Hence, these data show that p66^Shc^, in addition to regulating leukocyte transmigration, mediates also changes in endothelial cell permeability, through modifications in the VE-cadherin/β-catenin complex.

### Effects of p66^Shc^ silencing on TNFα-induced endothelial activation

**Figure 6:**
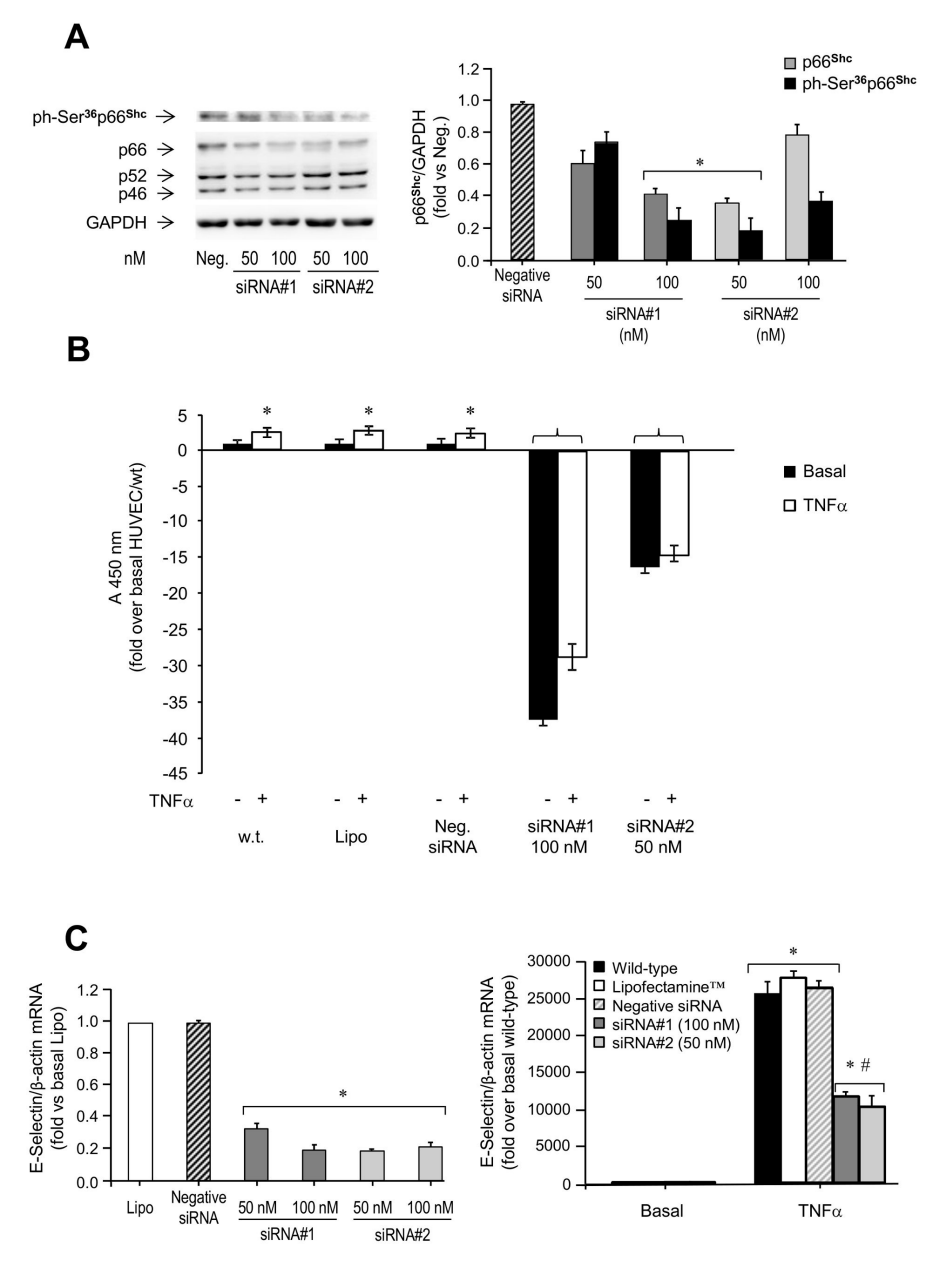
Effects of p66^Shc^ silencing on endothelial activation in HUVEC. *A*. (top left) Representative immunoblot of p66^Shc^ Ser^36^ phosphorylation in HUVEC transfected with 50 nM or 100 nM of siRNA#1 or siRNA#2, and (right) quantification of multiple experiments. *Hatched* *bar*, cells transfected with a negative control siRNA; black bars, cells treated with siRNA#1 and siRNA#2. (bottom left) Representative immunoblot of p66^Shc^ protein content in HUVEC transfected with 50 nM or 100 nM of siRNA#1 or siRNA#2, respectively, and (right) quantification of multiple experiments. *Hatched* *bar*, cells transfected with a negative control siRNA; grey bars, cells treated with siRNA#1; light grey bars, cells treated with siRNA#2. *P<0.05 vs. cells transfected with negative siRNA. Neg., negative siRNA. *B*. Leukocyte transmigration test. HL-60 cells were added to control HUVEC (w.t., Lipofectamine™, and negative siRNA) and HUVEC treated with p66^Shc^ siRNA#1 (100 nM) or p66^Shc^ siRNA#2 (50 nM), and left to migrate for 18 h at 37°C. Studies were carried out under basal conditions or following stimulation with 50 ng/ml TNFα for 4 h. Migrated cells were stained and measured by OD at 450 nm. Data represents the mean of triplicates from two independent experiments, and are normalized to basal HUVEC/wt. *P<0.05 vs. no TNFα; ^§^P<0.05 vs. HUVEC/wt. *C*. Effects of siRNA-mediated knockdown of p66^Shc^ on E-Selectin mRNA levels under basal conditions (left) and following TNFα stimulation (right). HUVEC were transfected with 100 nM siRNA#1 (grey bars) or 50 nM siRNA#2 (light grey bars), and then left untreated or incubated with 50 ng/ml TNFα for 1 h. *P<0.05 vs. basal; ^#^ *P*<0.05 vs. TNFα-stimulated controls (wild-type, Lipofectamine™, and negative siRNA). Lysates were analyzed by qPCR 48 h following transfection, using β-actin as internal control. Lipo, Lipofectamine™.

To confirm the involvement of p66^Shc^ in the effects of TNFα on endothelial activation, p66^Shc^ gene expression was silenced using two independent siRNAs. Transfection of HUVEC with these siRNAs resulted in 40% to 70% reductions, respectively, of p66^Shc^ mRNA levels compared to control (*P*<0.05; Figure S7A). p66^Shc^ protein levels and Ser^36^ phosphorylation were also significantly reduced with both siRNAs (*P*<0.05; Figure 6A).

Knockdown of p66^Shc^ resulted in a significant inhibition of leukocyte transmigration across the HUVEC monolayer (*P*<0.05; Figure 6B), and this was associated with 80% and 60% decreases of E-Selectin mRNA levels under basal conditions and following stimulation with TNFα for 1 h, respectively (*P*<0.05; Figure 6C). TNFα-induced E-Selectin protein expression was regulated through the JNK, but not ERK, pathway also in siRNA-treated HUVEC (Figure S7, B and C). These results indicate that p66^Shc^ plays an important role in leukocyte transmigration mediated by changes in E-Selectin expression in HUVEC.

### Involvement of oxidative stress in p66^Shc^ signaling

**Figure 7:**
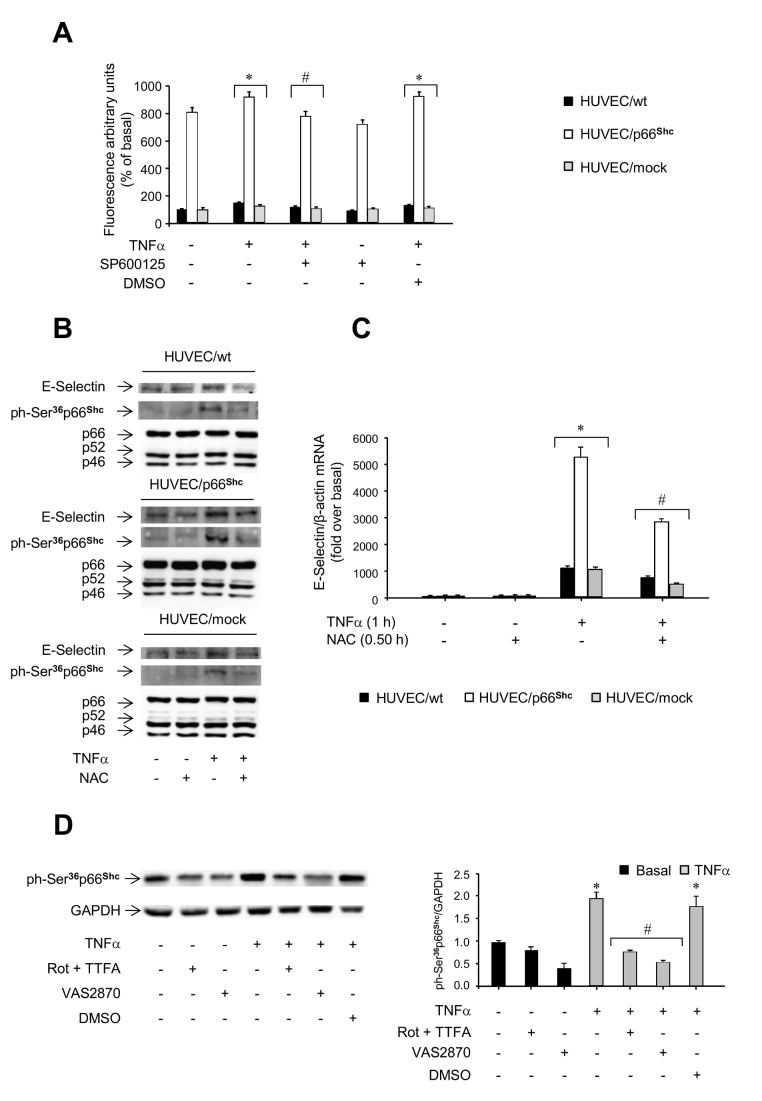
Role of oxidative stress in p66^Shc^ signaling. *A*. Levels of reactive oxygen species (ROS) in HUVEC/p66^Shc^ and control cells (HUVEC/wt and HUVEC/mock). Cells were pre-incubated with or without 30 mM SP600125 for 2 h and then treated with 50 ng/ml TNFα for 0.5 h or left untreated; ROS levels were evaluated by fluorimetry (HUVEC/wt, filled bars; HUVEC/mock, grey bars; HUVEC/p66^Shc^, open bars). *P<0.05 vs. untreated cells; ^#^ *P*<0.05 vs. cells stimulated with TNFα alone; all values of HUVEC/p66^Shc^ were significantly different (*P*<0.05) vs. HUVEC/wt. Data from multiple independent experiments (n=6) are expressed as mean±SE. *B*. Effects of N-acetyl-cysteine (NAC) on TNFα-induced phosphorylation of p66^Shc^ on Ser^36^. *C*. Effects of NAC on TNFα-induced E-Selectin expression. In *B* and *C*, HUVEC/wt (black bars), HUVEC/mock (grey bars), and HUVEC/p66^Shc^ (open bars) were preincubated with 10 mM NAC for 0.5 h before stimulation with 50 ng/ml TNFα. *P<0.05 vs. untreated cells; ^#^ *P*<0.05 vs. TNFα-stimulated cells not treated with NAC. *D*. Cells were coincubated with TNFα (50 ng/ml) and NADPH oxidase (Nox)-inhibitor 3-benzyl-7-(2-benzoxazolyl)thio-1,2,3-triazolo[4,5-d]pyrimidine (VAS2810, 5 µM) or rotenone (Rot, 10 µM) + thenoyltrifluoroacetone (TTFA, 10 µM) for 0.5 h. Representative immunoblots of p66^Shc^ phosphorylation on Ser^36^ and GAPDH (left), and ratio of phosphorylated p66^Shc^ protein to GAPDH (*right*; untreated cells, black bars; TNFα-treated cells, grey bars). *P<0.05 vs. basal; ^#^ *P*<0.05 vs. TNFα-stimulated cells.

To investigate whether p66^Shc^ regulates E-Selectin expression by modulating oxidative stress, intracellular reactive oxygen species (ROS) levels were measured in the experimental cells. In HUVEC/p66^Shc^, ROS levels were increased 8-fold compared to controls (*P*<0.05; Figure 7A). Stimulation with TNFα induced a weak but significant increase of ROS levels in control HUVEC and HUVEC/p66^Shc^ (*P*<0.05 vs. basal; Figure 7A), and pretreatment with SP600125 blocked this response (*P*<0.05 vs. cells treated with TNFα alone; Figure 7A). Furthermore, pretreatment with the antioxidant N-acetyl-cysteine (NAC) inhibited the increase in E-Selectin gene and protein expression induced by TNFα (Figure 7C), but also reduced p66^Shc^ phosphorylation on Ser^36^ (Figure 7B). In addition, coincubation of cells with TNFα and various ROS inhibitors, including the NADPH oxidase inhibitor VAS2870, and the complex I inhibitors rotenone and thenoyltrifluoroacetone (TTFA), respectively, resulted in reduced p66^Shc^ Ser^36^ phosphorylation induced by TNFα (Figure 7D). These results suggest that the involvement of p66^Shc^ in the TNFα-induced increase in E-Selectin expression is both dependent on and acting through an increase in intracellular ROS levels.

### Role of Ser^36^ phosphorylation of p66^Shc^ on leukocyte transmigration and E-Selectin gene expression, and endothelial permeability

To investigate the specific contribution of p66^Shc^ phosphorylation on Ser^36^ to the effects of TNFα, a mutant p66^Shc^ protein, in which Ser^36^ was replaced by Ala, was overexpressed in HUVEC (HUVEC/p66^Shc^ *Ala* ^*36*^). The adenovirus-mediated p66^Shc^ gene transfer augmented the protein levels of mutated p66^Shc^ by approximately 2-3 fold (Figure 4A); nevertheless, as expected, basal and TNFα-stimulated p66^Shc^ phosphorylation was similar in HUVEC/p66^Shc^ *Ala* ^*36*^ and in control cells, and was reduced compared to HUVEC/p66^Shc^ (Figure S4, *A-C*; Figure 4A). HUVEC morphology was not altered following overexpression of the mutant p66^Shc^ (Figure S4D). Importantly, HUVEC/p66^Shc^ *Ala* ^*36*^ showed a minimal increase in leukocyte transmigration compared to HUVEC/mock, but a markedly lower response compared to HUVEC/p66^Shc^ with total impairment of the TNFα effect (Figure 4B). Furthermore, E-Selectin mRNA levels were lower in HUVEC/p66^Shc^ *Ala* ^*36*^ than in HUVEC/p66^Shc^ and comparable to those found in HUVEC/wt and HUVEC/mock, both under basal conditions and after TNFα stimulation (Figure 4C). Similarly, HUVEC permeability to dextran was found to be lower in HUVEC/p66^Shc^ *Ala* ^*36*^ than in HUVEC/p66^Shc^ and not different from HUVEC/mock, both under basal conditions and following TNFα challenge (Figure 5). Therefore, p66^Shc^ phosphorylation on Ser^36^ appears to be important for the ability of this protein to mediate the effects of TNFα on E-Selectin gene expression and function in HUVEC, and this signaling event is apparently dependent upon JNK activation and ROS generation (Figure 4D). 

## Discussion

Endothelial dysfunction is a systemic disorder and a key variable in the pathogenesis of atherosclerosis and its complications. Expression of adhesion molecules, leading to leukocyte rolling and diapedesis, represents one of the earliest events in the atherogenic cascade. Hence, reverting the biological processes leading to endothelial activation may be an attractive target in the effort to optimize individualized therapeutic strategies to reduce cardiovascular morbidity and mortality [34] . The p66^Shc^ protein has been shown to control cellular responses to oxidative stress, being involved in atherosclerosis in animal models [26,35,36]. Deletion of the p66^Shc^ gene protects against age-related endothelial dysfunction [37] and reduces systemic and tissue oxidative stress, vascular cell apoptosis, and early atherogenesis in mice fed a high-fat diet [26]. In this study, we provide further insight into the cellular mechanisms mediating endothelial dysfunction, by showing that the stress-sensor protein p66^Shc^ is implicated in TNFα action in HUVEC, a well-defined cellular model of the human endothelium.

Accumulating evidence suggests that the pro-inflammatory cytokine TNFα plays an important role in the disruption of vascular responses [38]. TNFα stimulates the expression of endothelial cell genes, including E-Selectin, which may promote atherosclerosis via leukocyte recruitment [10]. In this study, challenge of HUVEC with TNFα resulted in increased E-Selectin expression, in line with similar results obtained in other endothelial cell systems [39]. This response occurred via the activation of the stress-kinase JNK [40], whereas ERK was apparently not involved. 

Phosphorylation of p66^Shc^ on Ser^36^ has been shown to mediate cellular aging, damage, and apoptosis, and to be induced by oxidative stress [41]. We found that stimulation of HUVEC with TNFα induced a 2- to 3-fold increase in p66^Shc^ phosphorylation on Ser^36^ (Figure 2, *A* and *B*), which was almost completely blocked by pretreatment of cells with the specific JNK inhibitor (Figure 2, C and D) and only partially reduced by pretreatment of cells with the p38 inhibitor (Figure S3A), in line with previous results obtained in bovine aortic endothelial cells exposed to H_2_O_2_ [41] and in HUVEC treated with the Vascular Endothelial Growth Factor [42]. Specific isoforms of the protein kinase C (PKC) family have also been proposed as mediators of ROS triggered stress signals. Indeed, oxidative stress has been shown to activate the PKC-β isoform, leading to Ser^36^-phosphorylation and mitochondrial translocation of p66^Shc^, and increased cell apoptosis [43]. In this study, the specific PKC-β inhibitor LY333531 reduced TNFα-induced phosphorylation of p66^Shc^ on Ser^36^ (Figure S3B). Interestingly, p66^Shc^ induces mitochondrial H_2_O_2_ production, resulting in further augmentation of intracellular H_2_O_2_ levels and PKC-β activation, in a sort of self-triggered control loop [43]. In HUVEC, the PKC-β inhibitor LY333531 reduced AGEs-induced macrophage adhesion to endothelial cells, thus relieving the local inflammation [44]. It has also been previously demonstrated that the inhibition of PKC-β prevents the activation of the JNK-p66^Shc^ proatherogenic pathway in human aortic endothelial cells [45], in line with the results of our study.

The effect of TNFα on p66^Shc^ phosphorylation on Ser^36^ was tightly associated with the ability of this cytokine to induce E-Selectin expression and leukocyte transmigration. Cells with selective overexpression of p66^Shc^ and enhanced p66^Shc^ phosphorylation on Ser^36^ showed increased E-Selectin expression and leukocyte transmigration, under basal conditions, and more so after stimulation with TNFα (Figure 3, B and C). A similar effect was observed when we studied endothelial permeability, which appeared to be increased in p66^Shc^-overexpressing cells, and further increased in response to TNFα (Figure 5F). This was associated with reduced VE-cadherin and increased β-catenin protein levels, respectively (Figure 5, *A-E*). Changes in VE-cadherin levels may significantly affect vascular permeability [16,22,46], and disruption of the adherent junctions at the level of VE-cadherin and β-catenin is an important mechanism leading to microvascular hyperpermeability [20]. Indeed, the increased degradation of VE-cadherin may be due to the disruption of cadherin–catenin complexes [47]. Interestingly, enhanced endothelial cell permeability associated with increased β-catenin expression and disruption of the VE-cadherin/β-catenin complexes, as found in HUVEC overexpressing wild-type p66^Shc^ (Figure 5) in this study, has been also observed in HUVEC treated with LPS and Ucn-1 [22].

Thus, p66^Shc^ appears to enhance the cellular sensitivity to the effects of TNFα, similarly to the increased susceptibility to toxic damage and apoptosis observed in other cellular models of p66^Shc^ overexpression [48]. Conversely, deletion of p66^Shc^ conferred protection from oxidative injury and apoptosis in both cellular systems *in vitro* and experimental animals *in vivo* [26,35]. In parallel with these observations, siRNA-mediated p66^Shc^ gene silencing resulted in marked reduction of E-Selectin expression and leukocyte transmigration both under basal conditions and after TNFα stimulation (Figure 6, B and C). The role of p66^Shc^ phosphorylation on Ser^36^ in this response was established by overexpressing a phosphorylation-defective p66^Shc^ mutant protein in HUVEC, which raised total p66^Shc^ protein levels to those found in the HUVEC overexpressing the wild-type p66^Shc^ (Figure S7, *A-C*; Figure 4A), but failed to enhance E-Selectin expression or leukocyte transmigration as that observed in HUVEC/p66^Shc^ (Figure 4, B and C). In multiple experimental models, p66^Shc^-dependent biological responses appear to be largely inhibited when Ser^36^ is mutated, even though the extent of inhibition may vary depending on the cell type and specific amino acid substitution (i.e., Ser-to-Ala vs. Ser-to-Asp) [49]. Our results confirm previous observations on the critical role of Ser^36^ phosphorylation of p66^Shc^ for induction of the apoptosis cascade in cells exposed to oxidative stress [24]. Interestingly, ROS production was associated with activation of the JNK-p66^Shc^ pathway (Figure 7A), and pretreatment with the antioxidant NAC resulted in a significant decrease in TNFα-induced p66^Shc^ phosphorylation and E-Selectin expression (Figure 7B). The mitochondrial respiratory chain is considered the main intracellular source of ROS; indeed, the NADPH oxidase complex has been involved in endothelial dysfunction [50] and Ser^36^ phosphorylation of p66^Shc^ in normal fibroblasts [51]. It was previously demonstrated that rotenone and TTFA (10 µM), which inhibit electron transfer to ubiquinone, significantly decreased ROS generation in HUVEC [52]. A decrease in ROS production has been reported also in HUVEC treated with the flavo-enzymes inhibitor diphenylene iodinium (10 µM) [52] and the NADPH oxidase inhibitor VAS2870 (5 µM) after incubation with TNFα and oxidized low-density lipoprotein, respectively [53,54]. In contrast, antimycin A (AA, 10 µM), a blocker of ubisemiquinone, increased ROS generation [52]. In this study, Ser^36^ phosphorylation of p66^Shc^ was significantly decreased when cells were exposed to the NADPH oxidase inhibitor VAS2810, and to the complex I inhibitors rotenone and TTFA, suggesting a role for both cytosolic and mitochondrial ROS in p66^Shc^ activation in endothelial cells (Figure 7D). Hence, E-Selectin expression appears to be, at least in part, under ROS control (Figure 7C), in line with previous reports [55]. In HUVEC/p66^Shc^, phosphorylation of p66^Shc^ on Ser^36^ was found to be significantly increased also in the absence of TNFα stimulation (Figure 4A), and this was associated with some enhancement of basal E-Selectin mRNA levels (Figure 3C). This “constitutive” p66^Shc^ Ser phosphorylation was partly reduced when HUVEC/p66^Shc^ were preincubated with the specific JNK inhibitor (Figure 4A), even though we could not document changes in JNK phosphorylation by immunoblotting (Figure S5A). This phenomenon could potentially result from the effects of augmenting p66^Shc^ protein levels in the presence of a low yet effective level of basal JNK activity.

The downstream targets of p66^Shc^, potentially involved in the regulation of E-Selectin gene expression in HUVEC, are still poorly characterized. p66^Shc^ has been shown to phosphorylate and inactivate FOXO3a, which is involved in the modulation of antioxidant defenses in cardiomyocytes [56]. However, there is no evidence of FOXO3a expression in endothelial cells, or of any FOXO3a-mediated transcriptional modulation of E-Selectin. p66^Shc^ has also been shown to regulate the transcriptional regulator Kruppel-like factor (KLF2), since knockdown of p66^Shc^ in endothelial cells induced mRNA expression of KLF2 and of its target gene thrombomodulin [57]. In addition, overexpression of KLF2 potently inhibited the induction of VCAM-1 and E-Selectin in response to proinflammatory cytokines, including TNFα [58], suggesting KLF2 as a likely candidate mediating the effects of p66^Shc^ on E-Selectin. However, we found that selective overexpression or knockdown of p66^Shc^ did not modify KLF2 mRNA levels in HUVEC (data not shown).

In animal models, the p66^Shc^ protein has been shown to be involved in the cellular events leading to atherosclerosis. Overexpression of the p66^Shc^ gene has been shown to reduce nitric oxide generation in endothelial cells, whereas downregulation or deletion of the p66^Shc^ gene improved the impaired endothelial cell-dependent vasodilation [36]. In humans, the expression of p66^Shc^ has been evaluated in circulating monocytes, which are key contributors to the vascular damage. The levels of p66^Shc^ in monocytes were significantly increased in patients with type 2 diabetes compared with controls, and were correlated to total plasma 8-isoprostane, a marker of oxidative stress [28]. More recently, p66^Shc^ was found to be increased in monocytes from patients with coronary artery disease, with a direct correlation between p66^Shc^ levels and the number of diseased vessels [59]. In this study, we provide evidence that p66^Shc^, through its regulatory Ser^36^ phosphorylation, lies downstream of JNK in the TNFα/JNK signaling pathway that controls E-Selectin gene expression in human endothelial cells (Figure 4D). Interestingly, modulation of p66^Shc^ expression appears to affect the ability of endothelial cells to induce leukocyte transmigration, a process which involves an E-Selectin-dependent mechanism [60] (Figure 1D), and Ser^36^ phosphorylation of p66^Shc^ is again essential for this phenomenon (Figure 4C). Thus, increased p66^Shc^ expression and activity may contribute to the endothelial dysfunction observed in individuals with high cardiovascular risk and potentially represent a novel marker of vascular injury.

## Supporting Information

Figure S1 **A.** **E-Selectin mRNA levels in siRNA-transfected HUVEC**. HUVEC were transfected with 50 nM or 100 nM of two different siRNAs specific for E-Selectin (siRNA#1 and siRNA#2). Cells were lysed after 48 h, and E-Selectin mRNA levels were measured by qPCR. siRNA Neg, cells transfected with a negative control siRNA. *B*. Time-course of TNFα-induced JNK-phosphorylation in HUVEC. Representative immunoblots of JNK phosphorylation (top left) and protein content (bottom left), and ratio of phosphorylated to total JNK proteins (*right*; JNK-1, grey *line*; JNK-2, *black* *line*). *C*. Time-course of TNFα-induced ERK phosphorylation. Representative immunoblots of ERK-1/2 phosphorylation (top left) and protein content (bottom left), and ratio of phosphorylated to total ERK-1/2 isoforms (*right*; ERK-1, black *line*, ERK-2, grey *line*).
(TIF)

Figure S2 **Effects of JNK and ERK inhibitors on TNFα-induced E-Selectin protein levels.** HUVEC were pre-treated with 30 mM of the JNK inhibitor SP600125 (Panel *A*) or the ERK inhibitor PD98059 (Panel *B*), respectively, for 2 h, and then challenged with 50 ng/ml TNFα for the indicated times (untreated cells, *black* *bars*; inhibitor-treated cells, *grey* *bars*; DMSO-treated cells, *light* *grey* *bars*). E-Selectin protein levels were evaluated by immunoblotting, using p66^Shc^ protein content as internal control. *C*. Representative images of HUVEC treated with the JNK inhibitor JNKi. Cells were treated with 10 mg/ml JNKi peptide linked to a FITC fluorochrome for 0.5 h, 1 h or 2 h, and analyzed by fluorescence microscopy. The green staining identifies peptide accumulation inside the cells (*left*). Cellular morphology was evaluated by optical microscopy (*right*). Representative images after 2 h of exposure to the JNKi are shown. *D*. Effects of JNKi on TNFα-induced c-Jun Ser^63^ phosphorylation and E-Selectin protein expression. HUVEC were preincubated with 10 mg/ml JNKi peptide for the indicated times, and then exposed to 50 ng/ml TNFα for 1 h. E-Selectin protein levels were evaluated by immunoblotting, using Shc protein expression as loading control. c-Jun Ser^63^ phosphorylation was evaluated as a readout for the activity of the inhibitor, using c-Jun protein content as loading control.
(TIF)

Figure S3 **Effects of p38 MAPK and PKC-β inhibition on Ser^36^ phosphorylation of p66^Shc^ in HUVEC.** *A*. Effects of the p38 MAPK inhibitor SB203580 on TNFα-induced phosphorylation of p66^Shc^ on Ser^36^. Cells were pre-treated with 15 or 30 µM SB203580 for 1 h and then exposed to 50 ng/ml TNFα for 0.5 h. Representative immunoblots of p66^Shc^ phosphorylation on Ser^36^ (*top left*) and Shc protein content (*middle left*), and ratio of phosphorylated to total p66^Shc^ protein (*right*; untreated cells, *black* *bars*; inhibitor-treated cells, *grey* *bars*). *B*. Effects of the PKC-β inhibitor LY333531 on TNFα-induced phosphorylation of p66^Shc^ on Ser^36^. Cells were pre-treated with 200 nM LY333531 for 1 h or 24 h and then exposed to 50 ng/ml TNFα for 0.5 h. Representative immunoblots of p66^Shc^ phosphorylation on Ser^36^ (*top left*) and Shc protein content (*middle left*), and ratio of phosphorylated to total p66^Shc^ protein (*right*; untreated cells, *black* *bars*; inhibitor-treated cells, *grey* *bars*). β-actin protein content was used as loading control. *P<0.05 vs. basal; ^#^ *P*<0.05 vs. TNFα. SB, SB203580; LY, LY333531.
(TIF)

Figure S4 **Overexpression of p66^Shc^ in HUVEC.** *A*. Representative immunoblots of p66^Shc^ phosphorylation on Ser^36^ and of Shc protein content in wild-type HUVEC (HUVEC/wt), HUVEC overexpressing p66^Shc^ (HUVEC/p66^Shc^), and HUVEC overexpressing p66^Shc^ with Ser^36^ to Ala mutation (HUVEC/p66^Shc^ *Ala* ^*36*^). HUVEC were infected with different PFU/ml of adenovirus, as indicated, and cell lysates were subjected to immunoblotting with specific antibodies. *B*. Quantification of p66^Shc^ protein content in HUVEC/p66^Shc^ (*black line*) and HUVEC/p66^Shc^ *Ala* ^*36*^ (*grey line*) under basal conditions. Protein content of p66^Shc^ is normalized to p52^Shc^ protein content; the p66 ^Shc^/p52^Shc^ ratio in wild-type cells was considered the reference value (PFU=0 in the graph). *C*. Ratio of basal p66^Shc^ Ser^36^ phosphorylation to total p66^Shc^ protein content in HUVEC/p66^Shc^ (*black line*) and HUVEC/p66^Shc^ *Ala* ^*36*^ (*grey line*), using the p66^Shc^ phosphorylation/content ratio in HUVEC/wt as reference (PFU=0 in the graph). *P<0.05 vs. controls (wild-type and mock). *D*. Representative images of HUVEC/wt, HUVEC/p66^Shc^, HUVEC/p66^Shc^ *Ala* ^*36*^, and HUVEC/mock. Cells were infected with the different adenoviral constructs and analyzed by fluorescent microscopy. The green staining identifies cells expressing the green fluorescent protein (GFP) encoded by the recombinant adenovirus. Cellular morphology was evaluated by optical microscopy.
(TIF)

Figure S5 **Phosphorylation of JNK and ERK and E-Selectin protein levels in HUVEC overexpressing p66^Shc^.** *A*. Basal and TNFα-stimulated phosphorylation of JNK1/2 in HUVEC overexpressing p66^Shc^. HUVEC/w.t., HUVEC/p66^Shc^ and HUVEC/mock were incubated with 50 ng/ml TNFα for the indicated times or left untreated. Cell lysates were subjected to immunoblotting with specific antibodies. Representative immunoblots of JNK1/2 phosphorylation and JNK1/2 protein content (*top*), and ratio of phosphorylated to total JNK proteins (*bottom*) (HUVEC/w.t. *black* *bars*, HUVEC/p66^Shc^, *open* *bars*; HUVEC/mock, *grey* *bars*). *B*. Basal and TNFα-stimulated phosphorylation of ERK1/2 in HUVEC overexpressing p66^Shc^. HUVEC/w.t., HUVEC/p66^Shc^ and HUVEC/mock were incubated with 50 ng/ml TNFα for the indicated times or left untreated. Cell lysates were subjected to immunoblotting with specific antibodies. Representative immunoblots of ERK-1/2 phosphorylation and protein content (*top*), and ratio of phosphorylated to total ERK-1/2 isoforms (*bottom*) (HUVEC/w.t. *black* *bars*, HUVEC/p66^Shc^, *open* *bars*; HUVEC/mock, *grey* *bars*). *P<0.05 vs. basal. *C*. Basal and TNFα-stimulated E-Selectin protein levels in HUVEC overexpressing p66^Shc^. HUVEC/p66^Shc^ and HUVEC/mock were incubated with 50 ng/ml TNFα for the indicated times or left untreated. Cell lysates were subjected to immunoblotting with specific antibodies. Representative immunoblots of E-Selectin protein levels (*left*), and the ratio of E-Selectin to GAPDH (used as loading control) protein levels in multiple experiments (*right*) are shown (HUVEC/p66^Shc^, *open* *bars*; HUVEC/mock, *grey* *bars*). *P<0.05 vs. basal; ^#^ *P*<0.05 vs. HUVEC/mock.
(TIF)

Figure S6 **Role of p66^Shc^ in TNFα regulated NF-κB pathway.** HUVEC/wt (*A*), HUVEC/mock (*B*), HUVEC/p66^Shc^ (*C*), and HUVEC/p66^Shc^ *Ala* ^*36*^ (*D*) were left untreated or incubated with TNFα for 15 min, and then analyzed by immunofluorescence. Adenovirus-infected cells are shown in green, NF-κB antibody staining is in red, and TOPRO-stained nuclei are in blue. The fuchsia staining results from the merging of red and blue, indicating NF-κB localization in the cell nucleus. *E*. Cell lysates were subjected to immunoblotting with ph-Ser^32^IκBα antibody using β-actin as loading control. Representative immunoblots (*left*) and the ratio of ph-Ser^32^IκBα to β-actin protein levels from multiple experiments (*right*) are shown (HUVEC/mock, *grey* *bars*, HUVEC/p66^Shc^ *Ala* ^*36*^ *black* *bars*, HUVEC/p66^Shc^, *open* *bars*). *P<0.05 vs. basal.
(TIF)

Figure S7 **siRNA-mediated knockdown of p66^Shc^.** *A*. p66^Shc^ mRNA levels in siRNA-transfected HUVEC. HUVEC were transfected with 50 nM or 100 nM of two different siRNAs specific for p66^Shc^ (siRNA#1 and siRNA#2). Cells were lysed 48 h or 72 h later, and p66^Shc^ mRNA levels were measured by qPCR. *wt*, wild-type cells; Lipo, cells treated with Lipofectamine™ only; Negative siRNA, cells transfected with a negative control siRNA. *B* and *C*. Effects of siRNA-mediated knockdown of p66^Shc^ on E-Selectin protein expression under basal conditions and following TNFα stimulation in HUVEC pretreated with SP600125 (*B*) and PD98059 (*C*). HUVEC were transfected for 72 h with 100 nM siRNA#1 and or negative control siRNA, and preincubated with SP600125 or PD98059 (30 mM, 2h), using DMSO as control, before treatment with 50 ng/ml TNFα for 1 h. Lysates were analyzed by immnoblotting, using p52^Shc^ and p46^Shc^ as controls. *Black* *bars*, cells transfected with a negative control siRNA; *white* *bars*, cells transfected with 100 nM siRNA#1.
(TIF)
