# Supplementary figures and images for: TNFα Signals via p66Shc to Induce E-Selectin, Promote Leukocyte Transmigration and Enhance Permeability in Human Endothelial Cells
Source: PLoS One. 2013 Dec 2;8(12):e81930. doi: 10.1371/journal.pone.0081930 (PMC3857848; doi:10.1371/journal.pone.0081930)

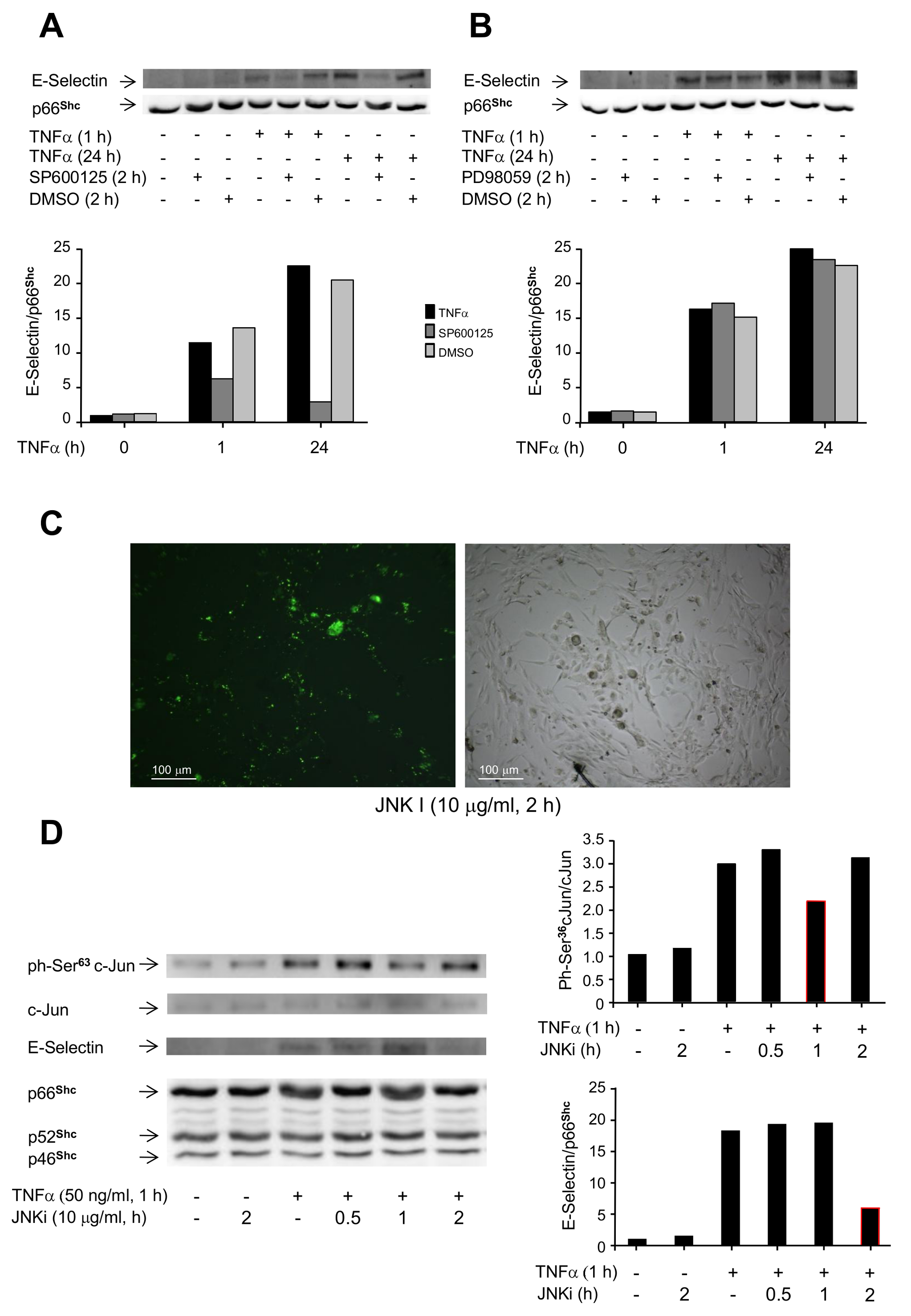

Supplement: Figure S2 — Effects of JNK and ERK inhibitors on TNFα-induced E-Selectin protein levels. HUVEC were pre-treated with 30 mM of the JNK inhibitor SP600125 (Panel A) or the ERK inhibitor PD98059 (Panel B), respectively, for 2 h, and then challenged with 50 ng/ml TNFα for the indicated times (untreated cells, black bars; inhibitor-treated cells, grey bars; DMSO-treated cells, light grey bars). E-Selectin protein levels were evaluated by immunoblotting, using p66Shc protein content as internal control. C. Representative images of HUVEC treated with the JNK inhibitor JNKi. Cells were treated with 10 mg/ml JNKi peptide linked to a FITC fluorochrome for 0.5 h, 1 h or 2 h, and analyzed by fluorescence microscopy. The green staining identifies peptide accumulation inside the cells (left). Cellular morphology was evaluated by optical microscopy (right). Representative images after 2 h of exposure to the JNKi are shown. D. Effects of JNKi on TNFα-induced c-Jun Ser63 phosphorylation and E-Selectin protein expression. HUVEC were preincubated with 10 mg/ml JNKi peptide for the indicated times, and then exposed to 50 ng/ml TNFα for 1 h. E-Selectin protein levels were evaluated by immunoblotting, using Shc protein expression as loading control. c-Jun Ser63 phosphorylation was evaluated as a readout for the activity of the inhibitor, using c-Jun protein content as loading control. (TIF) [file pone.0081930.s002.tif]

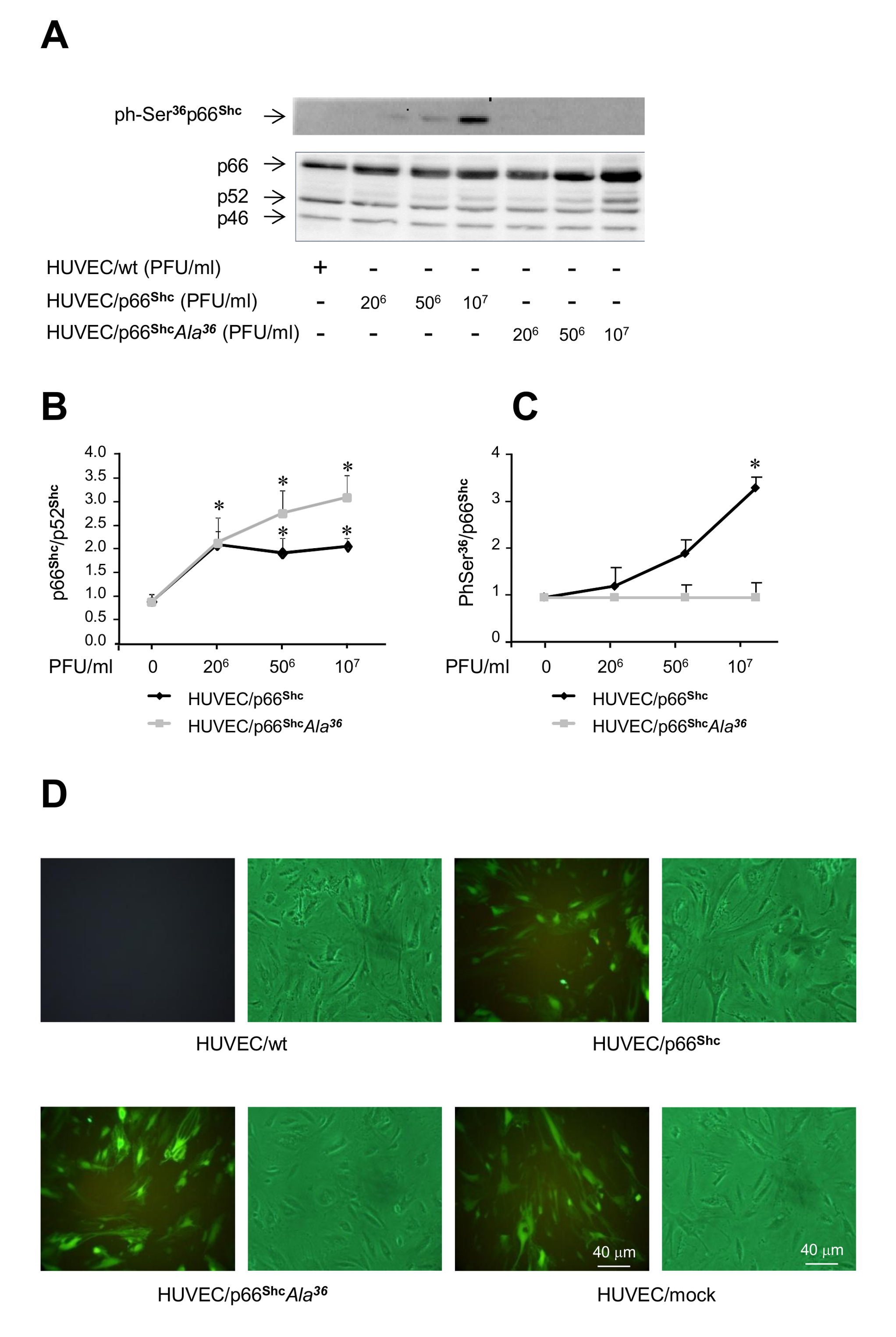

Supplement: Figure S4 — Overexpression of p66Shc in HUVEC. A. Representative immunoblots of p66Shc phosphorylation on Ser36 and of Shc protein content in wild-type HUVEC (HUVEC/wt), HUVEC overexpressing p66Shc (HUVEC/p66Shc), and HUVEC overexpressing p66Shc with Ser36 to Ala mutation (HUVEC/p66Shc Ala 36). HUVEC were infected with different PFU/ml of adenovirus, as indicated, and cell lysates were subjected to immunoblotting with specific antibodies. B. Quantification of p66Shc protein content in HUVEC/p66Shc (black line) and HUVEC/p66Shc Ala 36 (grey line) under basal conditions. Protein content of p66Shc is normalized to p52Shc protein content; the p66 Shc/p52Shc ratio in wild-type cells was considered the reference value (PFU=0 in the graph). C. Ratio of basal p66Shc Ser36 phosphorylation to total p66Shc protein content in HUVEC/p66Shc (black line) and HUVEC/p66Shc Ala 36 (grey line), using the p66Shc phosphorylation/content ratio in HUVEC/wt as reference (PFU=0 in the graph). *P<0.05 vs. controls (wild-type and mock). D. Representative images of HUVEC/wt, HUVEC/p66Shc, HUVEC/p66Shc Ala 36, and HUVEC/mock. Cells were infected with the different adenoviral constructs and analyzed by fluorescent microscopy. The green staining identifies cells expressing the green fluorescent protein (GFP) encoded by the recombinant adenovirus. Cellular morphology was evaluated by optical microscopy. (TIF) [file pone.0081930.s004.tif]

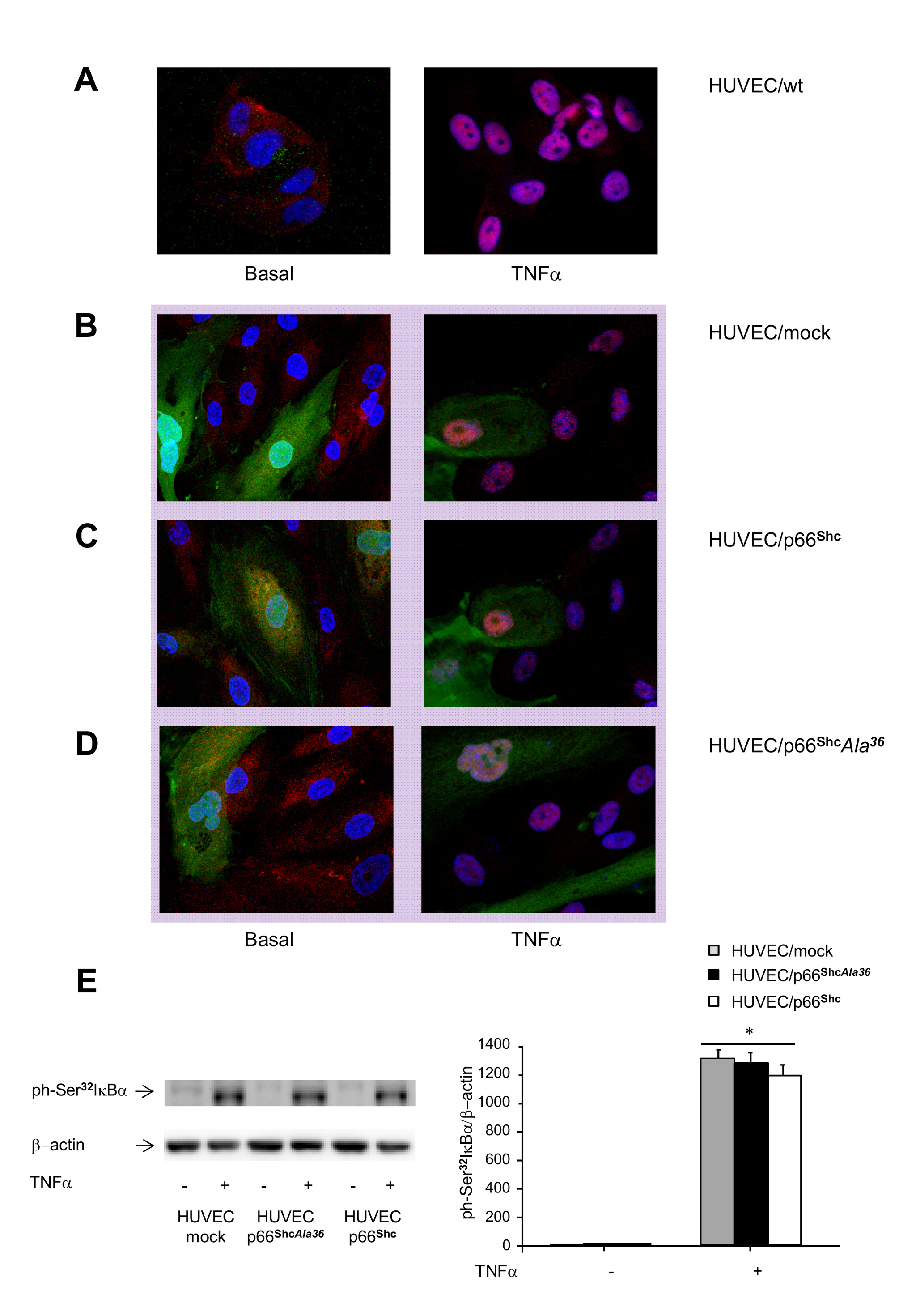

Supplement: Figure S6 — Role of p66Shc in TNFα regulated NF-κB pathway. HUVEC/wt (A), HUVEC/mock (B), HUVEC/p66Shc (C), and HUVEC/p66Shc Ala 36 (D) were left untreated or incubated with TNFα for 15 min, and then analyzed by immunofluorescence. Adenovirus-infected cells are shown in green, NF-κB antibody staining is in red, and TOPRO-stained nuclei are in blue. The fuchsia staining results from the merging of red and blue, indicating NF-κB localization in the cell nucleus. E. Cell lysates were subjected to immunoblotting with ph-Ser32IκBα antibody using β-actin as loading control. Representative immunoblots (left) and the ratio of ph-Ser32IκBα to β-actin protein levels from multiple experiments (right) are shown (HUVEC/mock, grey bars, HUVEC/p66Shc Ala 36 black bars, HUVEC/p66Shc, open bars). *P<0.05 vs. basal. (TIF) [file pone.0081930.s006.tif]
